# The effect of moisture content over the fibre saturation points on the impact strength of wood

**DOI:** 10.1098/rsos.231685

**Published:** 2024-02-28

**Authors:** Mojtaba Hassan Vand, Jan Tippner

**Affiliations:** Department of Wood Science and Technology, Faculty of Forestry and Wood Technology, Mendel University in Brno, Zemědělská 3, 613 00 Brno, Czech Republic

**Keywords:** wood, moisture content, impact test, three-point bending test, linden, fibre saturation point

## Abstract

The article's main aim is to assess the mechanical behaviour of linden under high-rate loadings (impact) and its change due to changes in moisture content (MC) over fibre saturation point. For assessing the mechanical properties of green wood, mainly the data of the dried wood is not applicable since the moisture content can drastically affect the mechanical properties of the wood. By testing both dried and high-moisture-content wood, we can understand a general viewpoint toward the effect of the moisture content on the impact behaviour of the wood. Several test samples were made of linden wood with different moisture content levels of 11%, 60% and 160%. A drop-weight impact machine tested the specimens to measure the reaction force of the hammer during a very short impact period. The results of the tests were parameters such as force–time chart, the maximum force required for crack initiation, the impact bending strength (IBS) and the work needed for crack initiation. The results indicated an increase in MC decreases the maximum force, work required for crack initiation and IBS drastically. However, when MC exceeded the fibre saturation point (FSP), there was no further influence on the force pattern and maximum required force.

## 1. Introduction

The article focuses on the impact behaviour of linden wood (*Tilia* × *europaea*), which is favourable for landscaping and urban forestry due to its benefits and unique characteristics which fit urban areas [1]. Enhancing our understanding of linden can significantly improve city safety and reduce accidents attributed to these trees. The main aim of this article is to determine the mechanical response of linden wood under the impact (high-rate loading) for moisture contents (MC) higher and lower than the fibre saturation point (FSP) to offer a general viewpoint toward assessing the linden wood as a dried building material and as a part of the tree. The effectivity of the MC at three different levels of MC was studied, and the relationship between the MC and the maximum force, the required work for crack initiation and the impact bending strength (IBS) of the linden were presented and analysed. These parameters can give a comprehensive viewpoint and serve as a complementary tool for assessing their structural integrity alongside other means of assessing the trees' strength.

The wood has evolved to fill the needs of the trees and many of these needs are mechanical, which are the need to support static and dynamic loads inflicted on the tree [2]. Besides the common properties of wood, there are essential properties of wood when loadings are a form of impact, such as impact toughness and energy absorbing capability [3]. Wood's remarkable ability to absorb energy made it suitable for many tasks regarding safe methods of handling the impacts, such as container production [4]. There are different methods of testing which measure the mechanical properties of wood related to impact, and one of them is the three-point bending impact test [5]. The impact test enforces a relatively high force of a hammer hitting the centre of the specimen during a few milliseconds to study the material's behaviour [6]. Impact tests can assess different sizes and conditions of the wood specimens, ranging from small-sized samples and veneers [7] up to actual-sized studs [8] and be used as a criterion alongside static tests for determining different affecting parameters [9] and reinforcing methods on wood to create improved products [10]. The importance of impact test of wood becomes more important progressively due to its application in modern industries for improving safety and the optimization of the design in the car industry [3,11], package industry [12] and the building industry [13,14]. Wood's ability to absorb energy by impact bending is called impact bending strength (IBS) and can be calculated using different testing methods [15] and be used as a criterion for assessing the strength of the wood against impacts and high-rate loadings [16]. However, this distribution of the absorbed energy is not uniform for wood, and the localized damage zone has the highest amount of absorbed energy and deformation [17]. This high level of deformation and absorption of the loading is due to the cellular or latticed structure of the wood, and deformation of the cells can endure significant compression loadings [18]. The strain distribution varies significantly, and normal compressive strain near the impact region is the highest, while the normal tensional strain is at the bottom of the central region of the beams, and the highest shear strain is on the neutral axis of wood [19]. The results of different tests may differ due to differences in methods and standards, making it challenging to compare the dynamic responses of different tests directly [20]. The main factors affecting the absorbed energy in impact bending are the viscosity and plasticity of wood [21]. Some testing settings can significantly affect the impact tests' results, such as impact velocity, inflicted energy and impactor physical appearance [22]. On the other hand, the material properties, such as the type of wood species, MC, temperature, the wood treatments, and presence of knots and defects, are also significantly effective [23–25].

Wood is a hygroscopic material, and the MC influences its various properties to different extents [26]. The MC of green wood of the linden is around 80% for heartwood and 130% for sapwood [27]. Around 30% MC, which is called ‘FSP', MC loses its influence on some properties of wood, and its change can no longer affect the wood's behaviour [28]. Both static properties of wood, such as Young's modulus and bending strength, and dynamic properties, such as the absorbed energy, depend on the MC of the specimens [29,30]. The impact strength can also be affected by the MC [31].

Small-leaved linden (*Tilia cordata* Mill*.*) and large-leaved linden (*Tilia platyphyllos* Scop.) are remarkably similar trees, native to Europe and prefer warmer climates, and their natural hybrid is known as common linden or European linden (*Tilia* × *europaea*) [32]. Linden is usually used to manufacture boxes and crates, wood turning and furniture manufacturing [33]. Several studies have focused on the assessment of the mechanical properties of linden wood under impact loading [34,35] and the affecting parameters on the impact strength, such as temperature [36], or with an aim to determine them for being used as a material properties input in finite-element method (FEM) [37].

## 2. Material and methods

### 2.1. Input production and preparation of the samples

Trunks of four linden trees were provided from a street in the centre of Brno, Czech Republic. It is worth mentioning that some studies showed environmental factors in urban regions can negatively impact tree growth and the wood quality of full-grown trees compared with remote regions [35]. Planks of wood were cut from the trunks, and the test specimens were made from these planks. The test samples were clear special orthotropic beams with dimensions of 300 × 20 × 20 mm without any knots and defects. Some research has indicated that impact tests require a higher number of test samples than static tests due to high variability in the results of the standard dynamic strength testing [38].

Ninety samples were prepared for impact test from boards made from different trees and divided into three groups of 30 samples. The first group (group A) was stored in an environment of 20°C and 65% relative humidity until the MC became stable verified by the specimens' periodic weighting. The second (group B) and third group (group C) specimens were submerged in water and then acclimatized above water in a closed compartment. These two groups had equilibrium moisture content (EMC) much higher than group A.

### 2.2. Impact tests configuration

**Figure 1.**
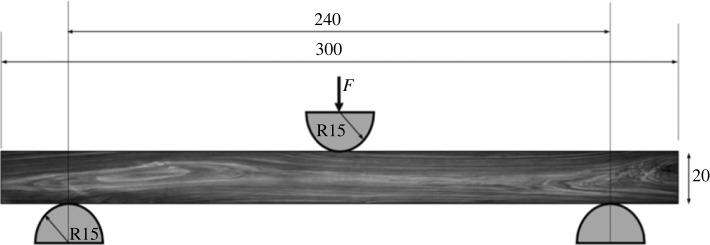
A schematic of dimensions of the test sample and clamps.

**Figure 2.**
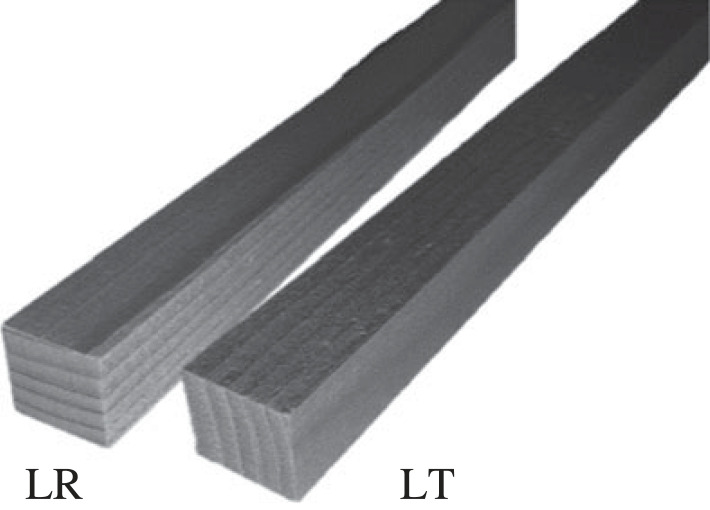
The orientation of the growth-ring under impact.

Impact tests were conducted by impact testing machine DPFest 400 (Labortech s.r.o., CZ) at the impact laboratory of Josef Ressel Research Centre (Mendel University in Brno, Útěchov, Czech Republic). A 9.05 kg hammer was released from 815.7 mm, and by reaching 4 m s^−1^ it inflicted 72.4 J energy on the wood samples. The readability of the position of the hammer was 0.01 mm, and a CFTplus force sensor located in the hammer itself measured the reaction force of the beam with a 1 MHz frequency. The temperature of the tests was the standard room temperature (approx. 20°C) and followed impact testing standards [39] and [40]. Figure 1 depicts the dimensions of the clamps of the testing machine. Each group was further divided into two subgroups based on the orientation of the growth rings of the samples. The loading was either parallel (tangential (LT)) or perpendicular (radial (LR)) to growth rings. Figure 2 shows both orientations of growth rings to the loading direction. The weight of the samples was measured just before testing. Also, after the tests, the samples were dried, and their dry weight was measured to calculate their MC during the test.

## 3. Results and discussion

**Figure 3.**
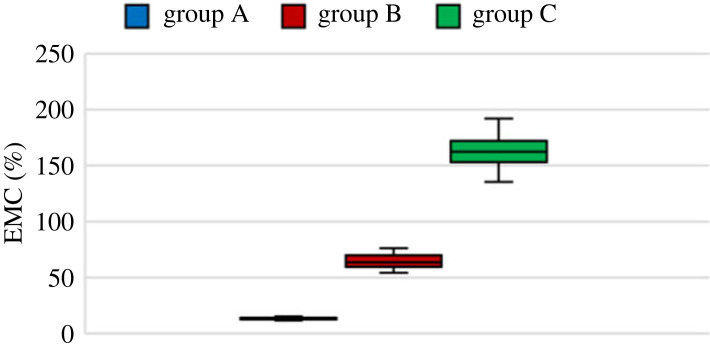
The EMC of the groups of samples.

The MC of all specimens was determined by measuring the weight of the specimens before and after the tests. Figure 3 depicts the EMC of all groups. The median of the MC of the groups was 13%, 64% and 160% for groups A, B and C, respectively.

**Figure 4.**
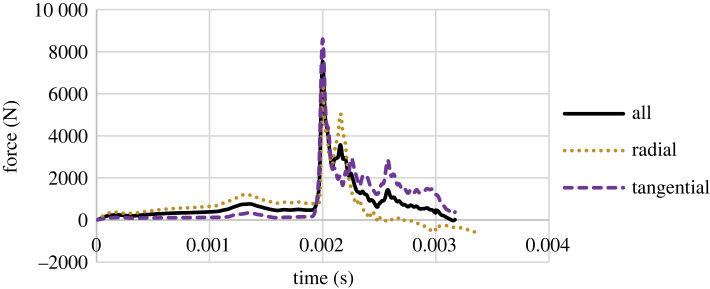
The force versus time chart for Group A samples in radial and tangential orientation.

**Figure 5.**
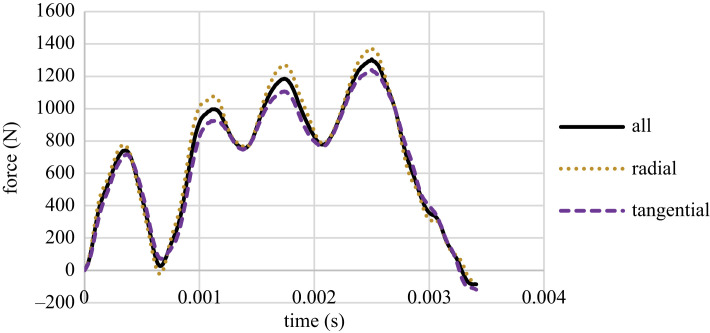
The force versus time chart for Group B samples in radial and tangential orientation.

**Figure 6.**
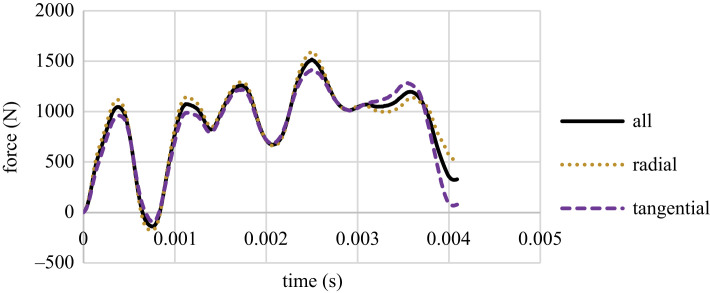
The force versus time chart for Group C samples in radial and tangential orientation.

The force–time charts are the output of the tests. There is only one point at which the peaks and troughs of the charts can cover each other due to the longevity of the charts; thus, for a correct pattern, there may be a need for a slight compression or stretching of the charts in the *x*-axis. Figures 4–6 present the three groups' median of force versus time charts. It was observed that the direction of growth-ring layers has no effect on the pattern of force–time charts, and the general pattern remains consistent, which was observed in previous studies [41]. However, the maximum force peak height in Group A differs for radial and tangential samples. These charts show that the assumption of lack of effect of the orientation of the layers on the result is applicable, especially for samples with higher MC.

**Figure 7.**
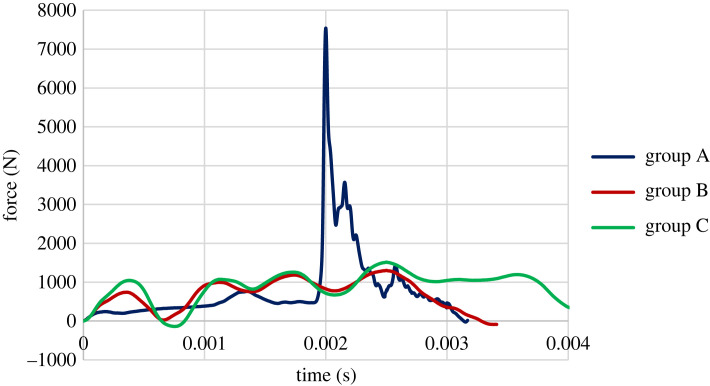
The force versus time charts of all groups.

Figure 7 compares these three groups' force versus time charts, depicting MC's effect on the material's behaviour. The inertia of the impact causes a local peak at the beginning of the chart, known as the inertial peak, which leads to a slight detachment of the specimen surface from the hammer, which shows itself as a local trough after the initial peak [42]. The damping effect of EMC increases the first peak of the chart. Group A's inertial peak and trough are much less than the other groups, possibly attributed to the difference in sample weight. For a high level of EMC, the charts become more damped with no sudden peak. Another crucial observation from this figure is that the difference between groups B and C is insignificant compared with Group A.

**Figure 8.**
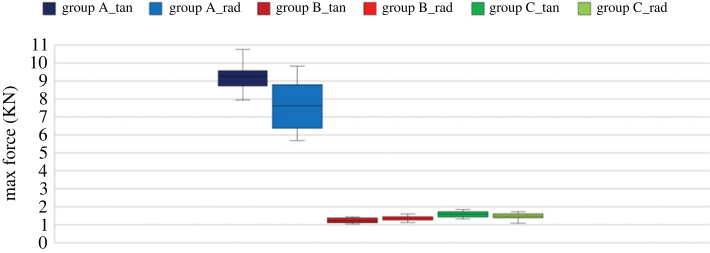
The boxplot chart of max force for both force orientation of EMC groups.

The maximum force is one of the criteria of the MC effect on the mechanical behaviour of the wood. Figure 8 displays the boxplot chart of the maximum force inflicted on the samples. It demonstrates that samples with low MC (Group A) exhibit a wide range of values, which indicates this group has high variability, unlike Groups B and C, which have a more concentrated boxplot of maximum force. Particularly, Group A's median stands out prominently when compared with the medians of the other groups with higher MC.

**Figure 9.**
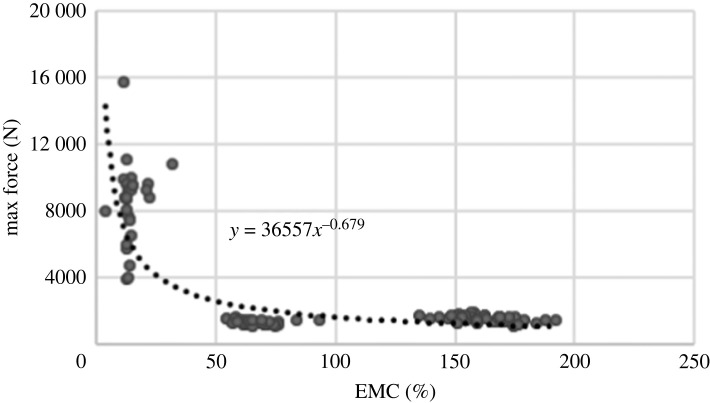
The scatter chart of maximum force of the impact.

A relationship pattern between maximum force and MC can be observed by plotting the maximum force versus MC scatter chart. The trend line of these scatter points is derivable. Figure 9 illustrates this scatter chart and its trendline. The groups can be easily diagnosed by being separated based on their EMC.

**Figure 10.**
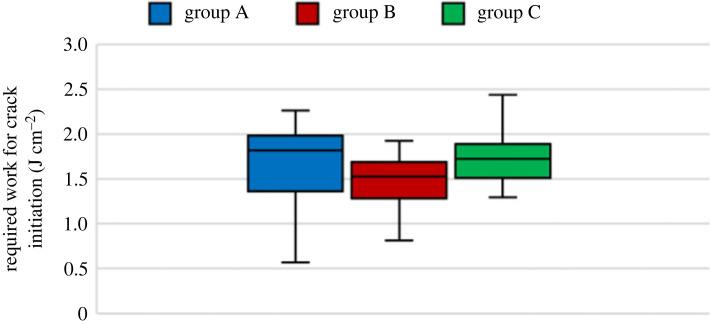
Required work for crack inititaion of all EMC groups.

**Figure 11.**
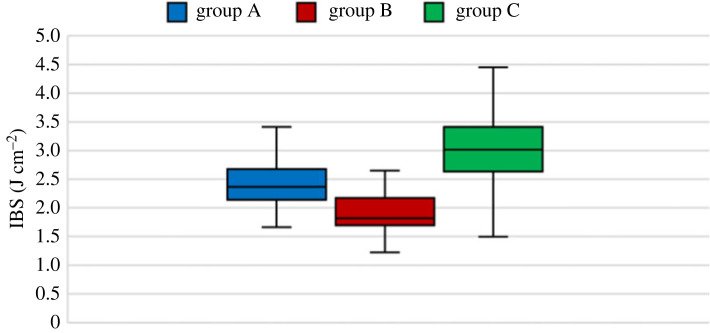
IBS of all groups.

**Figure 12.**
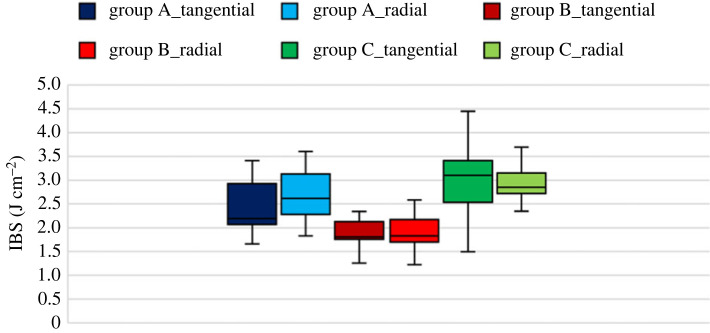
IBS of all groups for both growth ring directions.

Other important parameters for assessing MC's effect on Linden's impact strength are the work required for crack initiation and the total work for rupture or IBS. IBS is more crucial in determining the material's strength and effectiveness than maximum force since the maximum force can appear as a narrow force pulse [41]. Table 1 and figures 10–12 show these parameters, respectively. The ANOVA test was done between the groups to determine if the MC could affect the required work for crack initiation, and it was observed that MC has no significant effect on the work for crack initiation since the *p*-value of the test for group A and others is more than 0.05, and the null hypothesis is correct. However, for the IBS, the *p*-value of the *T*-test was much less than 0.05, leading to the rejection of the null hypothesis, which indicates that MC has an apparent effect on the IBS.

**Table 1.** The work required for crack initiation and rupture for all EMC groups.

	Group A	Group B	Group C
work for crack initiation (J cm^−2^)	1.82	1.53	1.88
IBS (J cm^−2^)	2.37	1.82	3.01

By comparing the results of this article with other research, we can see that the IBS of linden was determined to be 6.5 J cm^−2^ [23] and 1.9 J cm^−2^, which is 176% and 20% different from our results, respectively.

## 4. Conclusion

By considering the results of this article as a piece of collective information, we can grasp a general viewpoint toward the behaviour of wood under impact and the effect of the MC on its strength.

The loading orientation has a negligible effect on the impact strength and force–time pattern. This means that wood can be assumed to behave as transverse isotropic material under impact loading.

By increasing EMC, the behaviour of the linden tends to reach a stable pattern in a way that for MC higher than FSP, the change in the force pattern would be negligible. This conclusion would apply to the investigation of the behaviour of wood under impact for EMCs higher than FSP by generalizing a pattern for all levels of MC.

The EMC effectively decreases the maximum force required for crack initiation. However, the maximum force for lower EMC is a peak in the form of a narrow pulse. Also, the range of maximum force severely decreases with increased MC.

The moisture content does not affect the work for crack initiation since the ANOVA test of the three groups could not reject the null hypothesis. On the other hand, MC has an apparent effect on the IBS; however, the effect is unclear.

The results of this research have an excellent potential to be used alongside other methods of measurements and modelling, such as the digital image correlation (DIC) method for assessing the deflection and strain pattern or the FEM for modelling of the linden wood in FEM software. Combining these approaches can offer a comprehensive viewpoint of linden wood's mechanical behaviour.

## Data Availability

The data are provided in electronic supplementary material [43].
